# Intensive sequential tDCS–rTMS over left DLPFC for biperiden dependence: A case report

**DOI:** 10.3934/Neuroscience.2026021

**Published:** 2026-08-20

**Authors:** Antonio Romeo, Antonio Bruno, Maria Rosaria Anna Muscatello, Fabrizio Turiaco

**Affiliations:** 1 Department of Biomedical and Dental Sciences and Morphofunctional Imaging, University of Messina, Messina, Italy; 2 Psychiatry Unit, Polyclinic Hospital University of Messina, Messina, Italy

**Keywords:** biperiden dependence, tDCS, rTMS, left DLPFC, neuromodulation

## Abstract

**Background:**

Anticholinergic misuse is an underrecognized form of substance use disorder that can be difficult to treat. Noninvasive neuromodulation targeting prefrontal control networks has shown promise across substance use disorders but has not been described for biperiden dependence.

**Objective:**

To report feasibility, tolerability, and preliminary clinical outcomes of a brief, intensive combined neuromodulation protocol; sequential anodal transcranial direct current stimulation (tDCS) followed by high frequency repetitive transcranial magnetic stimulation (rTMS) to the left dorsolateral prefrontal cortex (lDLPFC) in a single patient with refractory intramuscular biperiden dependence.

**Case and Methods:**

A 38-year-old man with schizoaffective disorder and borderline personality disorder self-administered up to 15 mg/day of intramuscular biperiden. He underwent an intensive course of neuromodulation consisting of two daily sessions (separated by a 20 minute break) for five consecutive days; each session comprised anodal tDCS to the lDLPFC (1.5 mA; 20 min 6 s) immediately followed by high frequency rTMS to the lDLPFC (15 Hz; 2400 pulses/session; 100% motor threshold). Addictive behavior was assessed with the Addictive Behavior Questionnaire (ABQ, 7DAS subscale) at baseline (T0), end of treatment (T1), and one month follow up (T2); daily vial consumption was recorded.

**Results:**

The 7DAS score decreased from 51 at T0 to 38 at T1, with a partial rebound to 46 at T2. Reported vial consumption fell from ≈3/day at baseline to 0/day by the end of treatment and remained between 0 and 1/day at one month. The intervention was well tolerated; only transient scalp discomfort and mild, short lived headaches were reported.

**Conclusions:**

In this single case, a brief intensive sequential tDCS→rTMS protocol was feasible, well tolerated, and temporally associated with rapid reduction in biperiden use and improvement in addictive behavior scores. Findings are preliminary and hypothesis generating; controlled studies with objective toxicology, standardized stimulation parameters, and longer follow up are needed to evaluate efficacy, mechanisms, and durability.

## 1. Introduction

According to the Diagnostic and Statistical Manual of Mental Disorders (DSM-5-TR), substance use disorder (SUD) is a condition characterized by the compulsive use of a substance with negative social, occupational, or personal consequences [1]. This unified category replaced the DSM-IV-TR distinctions between substance abuse and substance dependence and encompasses symptoms that reflect loss of control, compulsive use, persistence despite harm, and neuroadaptive changes [2],[3]. On a neurobiological level, SUD mainly involves the reward system, in particular the mesocorticolimbic dopamine circuits, which are responsible for regulating reward, motivation, and reinforcement [4]. Chronic exposure to the substance alters dopaminergic signaling mechanisms and functional connectivity within dopaminergic pathways, inducing aberrant brain responses and neuroadaptive phenomena that account for tolerance and withdrawal syndrome. Furthermore, dysfunctions are observed in the prefrontal circuits, responsible for inhibitory control and planning, which contribute to the loss of behavioral control, a core feature of the disorder [5]. Biperiden and other anticholinergic agents can ameliorate negative psychotic symptoms and reduce neuroleptic induced anhedonia, inducing psychotic patients to abuse them. Mesolimbic dopaminergic system, formed by the ventral tegmental area, the nucleus accumbens, and the prefrontal cortex is deeply involved in development drug abuse [6]. On one hand, anticholinergic drugs can inhibit M4 receptors, determining dopamine release in the nucleus accumbens, reducing emotional blunting and loss of interest, and increasing motivational drive. On the other hand, the blockade of M1 receptors, especially at the cortical and hippocampal levels, can generate subjective experiences of dissociation or altered states of consciousness that some individuals find desirable [7].

Current treatments combine pharmacological approaches with psychotherapy, mainly pure cognitive or mixed cognitive-behavioral models, for promoting abstinence, and for addressing symptoms of withdrawal, craving, and the related symptomatic clusters of negative emotional states, dysphoria, depression, sleep problems, cognitive impairment, and social isolation [8].

Beyond traditional treatment strategies, and within a dimensional approach, neuromodulation may be suitable for improving symptoms associated with substance use disorders, either in the acute phase of withdrawal and in maintaining abstinence, addressing more protracted affective symptoms and disrupted sleep patterns. Transcranial magnetic stimulation (TMS) and transcranial direct current stimulation (tDCS) may be promising brain-based techniques since they modulate key neurocircuits involved in reward processing, executive control, and craving; furthermore, they may also be considered as augmentation strategies for those patients who do not respond to available therapies [9]–[12]. Nevertheless, extended treatment schedules require considerable time and commitment, and this can result in low adherence or in organizational difficulties that compromise therapy completion.

Biperiden is an anticholinergic agent widely used to treat extrapyramidal symptoms associated with antipsychotic therapy; however, misuse and dependence have been reported in the literature. Case reports and reviews describe patterns of dose escalation, compulsive use, cognitive impairment, delirium, and functional decline among patients who misuse anticholinergics, often in the context of psychotic disorders, polysubstance use, or social marginalization. Evidence is limited and mostly consists of case reports and small series, underscoring that biperiden dependence is uncommon but clinically important and likely underrecognized [13],[14].

To improve adherence to treatment, we applied a brief, intensive 5-day combined tDCS and rTMS protocol designed to target refractory biperiden dependence. The sequential use of tDCS as a cortical primer followed by frequency-dependent rTMS aims to enhance synaptic plasticity and produce more robust, targeted neuroplastic changes than either technique alone. The tDCS increases cortical excitability, inducing a neuronal subthreshold depolarization. This could facilitate LTP-like plasticity through enhanced NMDA receptor activation and calcium influx, making tDCS a cortical priming intervention for subsequent rTMS stimulation [15],[16].

## 2. Case presentation

D. is a 38-year-old man with a diagnosis of schizoaffective disorder and borderline personality disorder. There is no reported family history of psychiatric illness. Symptom onset dated to age 14 with dysphoria, social withdrawal, and uncontrolled emotional outbursts often resulting in aggressive behavior. Between ages 15 and 16, he was hospitalized twice in the Child and Adolescent Neuropsychiatry unit; since then, he has had multiple voluntary and involuntary admissions, the most recent in November 2023. His history is notable for poor adherence to prescribed treatments and episodes of self-medication. Over time, he received several antipsychotics and mood stabilizers.

The patient presented with an unmonitored, escalating intramuscular biperiden intake meeting criteria for a substance use disorder. He had been dependent for at least one year and reported self-administering up to 15 mg intramuscular biperiden per day (three vials/day).

Because of refractory addictive behavior and limited response to prior interventions, an intensive combined neuromodulation protocol was proposed as an adjunctive treatment. The risks, potential benefits, and off label nature of the intervention were discussed with the patient, and written informed consent was obtained. The local ethics committee was notified, and the procedure was performed in accordance with institutional policies for off label neuromodulation. Adverse events were monitored throughout.

The intensive protocol comprised two daily sessions for five consecutive days. Each daily session consisted of tDCS followed in sequence by rTMS; the two sessions were separated by a 20-minute break. Stimulation sites were localized using craniometric landmarks (no MRI based neuronavigation). The Beam F3 method was used for the localization of lDLPFC. The program (http://clinicalresearcher.org/eeg/) provides instructions for finding the F3 location from the International 10–20 system. First, the distance from nasion to inion was measured, marking the halfway point on this line. Then, the measurement from the left preauricular point to the right preauricular point was taken, marking the halfway point on this line and the vertex. Finally, the circumference was measured. Two output values emerged: The distance (point-x) along the circumference from the centerline and the distance from the vertex along a line intersecting point-x. The distance from the vertex indicated by the computer program along a ray beginning at the vertex and intersecting point-x was the F3 location [17]. The patient remained on valproic acid 300 mg twice daily, olanzapine 20 mg/day, and lorazepam 2.5 mg twice daily throughout the 5-day protocol.

For tDCS, the following parameters were used: PiSTIM circular electrodes (reported diameter 12 mm; reported surface area 3.14 cm²) were positioned over the DLPFC regions. Anodal stimulation of the left DLPFC (lDLPFC) was delivered at 1500 µA (1.5 mA) with the cathode over the right DLPFC (rDLPFC). A high-conductivity saline gel was applied to each electrode. Stimulation duration was 20 minutes and 6 seconds per tDCS application.

Resting motor threshold (RMT) was determined over the primary motor cortex (PMC) as the minimum stimulation intensity able to evoke motor evoked potentials (MEP) in the contralateral abductor pollicis brevis (APB) in at least 5 of 10 trials. Immediately after each tDCS application (after a brief pause), rTMS was applied to the lDLPFC using a high-frequency protocol: 15 Hz, 2400 pulses per session delivered as 50 pulses/train across 48 trains, with a 23-second inter-train interval, at 100% of the resting motor threshold. Each day, the tDCS→rTMS sequence was performed twice.

Addictive behavior was assessed with the Seven Domains Addiction Scale (7DAS) of the Addictive Behavior Questionnaire (ABQ). The 7DAS evaluates the core psychological vulnerabilities that maintain addictive behaviors. It uses a 5-point Likert scale across seven subscales: Separation anxiety, Affect dysregulation, Somatoform and psychological dissociation, Childhood traumatic experiences, Impulse dyscontrol, Compulsive behavior and ritualization, and Obsessive thoughts. Higher mean subscale scores indicate a greater presence of the corresponding psychological trait or vulnerability [18]; moreover, the daily vial consumption was recorded. Assessments were performed at baseline (T0), end of treatment (T1), and one-month follow-up (T2). Adverse events and tolerability were recorded at each session.

## 3. Results

At baseline, the ABQ 7DAS score indicated moderate addictive behavior (T0 = 51). At the end of treatment, the 7DAS score decreased (T1 = 38). At one month, the score showed a partial rebound (T2 = 46) (Figure 1). Objective consumption data showed a reduction from approximately 3 vials/day at baseline to 0 vials/day by the end of the 5-day course; at one month post-treatment, consumption ranged between 0 and 1 vial/day. No serious adverse events occurred; the patient reported transient scalp discomfort during rTMS and occasional mild, short-lived headache after some sessions, both resolving without intervention.

**Figure 1.**
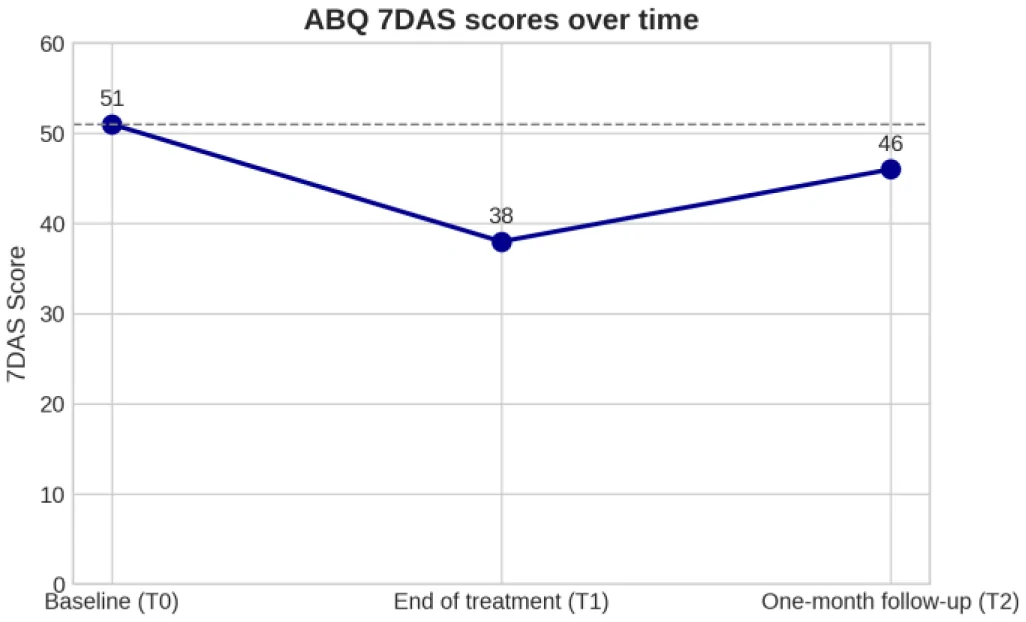
ABQ 7DAS scores at baseline (T0), end of treatment (T1), and one-month follow-up (T2). Scores decreased at T1 with a partial rebound at T2.

## 4. Discussion and conclusion

In this single case of refractory intramuscular biperiden dependence, a brief intensive neuromodulation protocol combining sequential anodal tDCS and high frequency rTMS over the left dorsolateral prefrontal cortex (lDLPFC) was associated with a marked short-term reduction in self-reported addictive behavior (ABQ 7DAS: 51 → 38) and an immediate cessation of reported vial use by the end of the 5 day course (≈3 vials/day → 0). At one month follow up, there was a partial rebound in the 7DAS score (46), while objective consumption remained substantially lower than baseline (0–1 vials/day). The intervention was well tolerated, with only transient scalp discomfort and mild headaches reported.

Interestingly, the partial rebound in ABQ scores at one-month follow-up contrasted with the sustained reduction in biperiden consumption. This dissociation may suggest that the sequential tDCS-rTMS intervention primarily enhanced executive functioning and inhibitory control over drug-seeking behavior, enabling the patient to limit substance use despite the persistence of some subjective craving.

The observed clinical changes are consistent with the hypothesized role of prefrontal circuits in exerting top-down control over compulsive drug seeking and craving. High-frequency rTMS to the lDLPFC is thought to enhance cortical excitability and strengthen executive control networks, while anodal tDCS may prime cortical excitability and facilitate subsequent TMS-induced plasticity. Together, the sequential tDCS→rTMS approach may have produced synergistic modulation of prefrontal–striatal–limbic circuits, reducing craving and improving inhibitory control sufficiently to interrupt habitual biperiden self-administration. Additional mechanisms specific to anticholinergic agents are speculative but could include indirect modulation of cholinergic–dopaminergic interactions that influence reward salience and cognitive control.

Although the partial rebound in ABQ score at one month highlights the uncertain durability of effects after a short intensive course, this case suggests that a brief, intensive combined tDCS→rTMS protocol may be a feasible adjunctive option for selected patients with refractory anticholinergic dependence, particularly when rapid reduction of use is clinically desirable.

Given the uncontrolled design and single subject nature of the observation, these results are preliminary and hypothesis generating. Controlled trials with objective outcome measures and longer follow up are required to establish efficacy, optimal parameters, and long-term safety of neuromodulation for anticholinergic substance use disorders.

## Use of AI tools declaration

The authors declare they have not used Artificial Intelligence (AI) tools in the creation of this article.
